# Role of Seaports and Imported Rats in Seoul Hantavirus Circulation, Africa

**DOI:** 10.3201/eid2901.221092

**Published:** 2023-01

**Authors:** Guillaume Castel, Claudia Filippone, Caroline Tatard, Jacques Vigan, Gauthier Dobigny

**Affiliations:** CBGP, INRAE, CIRAD, IRD, Institut Agro, University of Montpellier, Montpellier, France (G. Castel, C. Tatard, G. Dobigny);; European Research Infrastructure on Highly Pathogenic Agents, Bruxelles, Belgium (C. Filippone);; National University Hospital Center, Cotonou, Benin (J. Vigan);; Institut Pasteur de Madagascar, Antananarivo, Madagascar (G. Dobigny)

**Keywords:** Seoul hantavirus, viruses, zoonoses, vector-borne infections, orthohantavirus Seoul, zoonotic emergence, rodent-borne infections, *Rattus rattus*, *Rattus norvegicus*, urbanization, seaport, international trade, surveillance, spillover, Africa

## Abstract

Seoul orthohantavirus (SEOV) is not considered a major public health threat on the continent of Africa. However, Africa is exposed to rodentborne SEOV introduction events through maritime traffic after exponential growth of trade with the rest of the world. Serologic studies have already detected hantavirus antibodies in human populations, and recent investigations have confirmed circulation of hantavirus, including SEOV, in rat populations. Thus, SEOV is a possible emerging zoonotic risk in Africa. Moreover, the range of SEOV could rapidly expand, and transmission to humans could increase because of host switching from the usual brown rat (*Rattus norvegicus*) species, which is currently invading Africa, to the more widely installed black rat (*R. rattus*) species*.* Because of rapid economic development, environmental and climatic changes, and increased international trade, strengthened surveillance is urgently needed to prevent SEOV dissemination among humans in Africa.

Rodents are widespread, opportunistic, and competent host reservoirs involved in the maintenance, circulation, and transmission of a wide panel of zoonotic pathogens (*1*). Rodent-related zoonoses cause up to 400 million human infections worldwide each year (*1*,*2*). Among zoonotic pathogens, hantaviruses (order Bunyavirales, family Hantaviridae, genus *Orthohantavirus*) are among agents considered most likely to emerge and have a global public health impact (*3*). 

Hantaviruses are enveloped, negative, single-stranded RNA viruses with a tripartite genome comprised of large, medium, and small segments. Transmitted to humans via inhalation of aerosolized virus in contaminated rodent urine and feces, hantaviruses can cause hemorrhagic fever with renal syndrome (HFRS) or hantavirus pulmonary syndrome (*4*). Hantaviruses are generally carried by a rodent species host, and geographic distribution of the host can determine the area in which the associated disease occurs among humans. From this perspective, Seoul orthohantavirus (SEOV), identified in South Korea in 1982, deserves special attention because its cosmopolitan host, the Norwegian rat (*Rattus norvegicus*), also known as the brown rat, has been dispersed worldwide, resulting in a global distribution of the virus today (*5*). Detection of SEOV is often considered anecdotal and speculated to be driven by sporadic introduction of infected brown rats via transportation but also by pet or laboratory rats (*6*,*7*). Diagnosing SEOV in humans remains a challenge due to milder and atypical HFRS pathology (*8*). However, mild symptoms can progress to acute renal disease associated with HFRS, in which patients experience low blood pressure, acute shock, and acute kidney failure, and the case-fatality rate is ≈1% (*9*). 

## History of Hantaviruses in Africa

Fifteen years ago, no indigenous hantavirus was known in Africa (*10*). Since then, few studies have investigated hantaviruses, including SEOV, in Africa and consequences for human health. The dearth of studies gives the appearance that SEOV is not a major public health threat on the continent because of the lack of local specific testing for SEOV among human serum samples (*11*). Nonetheless, suspicions of SEOV-like agents in humans and wild rats in 17 different countries in Africa are strong (*5*). Until recently, immunofluorescence assays positive for Hantaan virus (HTNV), a closely related orthohantavirus in rats, was the only indication that SEOV probably was in Africa. Unfortunately, these serologic analyses were mainly based on cross-reactivity with better documented hantaviruses from Eurasia within the *Murinae*-associated hantavirus virus genera and did not enable identification of viruses at a finer specific level (*12*). In addition, these analyses usually lacked confirmatory assays (*13*). However, because of the high specificity of hantaviruses for their rodent hosts, positive serologic tests in rats could be ascribed to cross-reactions with SEOV or SEOV-like variants (*5*), as seen in Senegal (*12*,*13*). Of note, older serologic studies in Africa, including regions in West Africa, have detected antibodies against hantaviruses in the human general population and in febrile patients with putative hantavirus disease (*13*–*15*). Detecting putative hantavirus in febrile patients is a crucial public health issue in Africa, where fever of unknown etiology is very common. However, in the absence of differential diagnosis and further laboratory confirmation, we cannot be certain of the virus involved in these cases. We also cannot consider these initial observations exhaustive because of the lack of a proper epidemiologic approach and the limits of the methods used. Nonetheless, those reports might represent a primordial reflection of the health effects that hantavirus zoonoses could have in Africa. 

Since 2006, a genus-reactive pan-hantavirus PCR has been available to search for new hantaviruses in small mammals (*16*,*17*). This PCR led to the discovery of the 2 molecularly characterized endemic hantaviruses in mammals in Africa: Sangassou virus in the African wood mouse (*Hylomyscus simus*) and Tanganya virus in the Therese’s shrew (*Crocidura theresae*) (*13*,*16*,*17*). Since those discoveries, up to 10 indigenous hantaviruses have been identified in rodents, shrews, and even bats in Africa, making it the continent with the most recent scientific progress in hantavirus epizootiology and epidemiology (*10*). Recently, 2 studies using the pan-hantavirus PCR have molecularly assessed SEOV in rodents from southeastern Senegal (*18*) and southern Benin (*19*), confirming that SEOV circulates in West Africa and could be a cause of hantavirus disease in humans (Figure). In both cases, phylogenic analyses grouped the retrieved viral sequences with SEOV strains from Asia but from 2 different genetic lineages (*19*). Strains from Benin belonged to SEOV lineage 7, whereas lineages from Senegal belonged to SEOV lineage 3 or 4, depending on the genomic segment considered (Figure); this difference could indicate different introduction events in these 2 countries (*19*).

**Figure:**
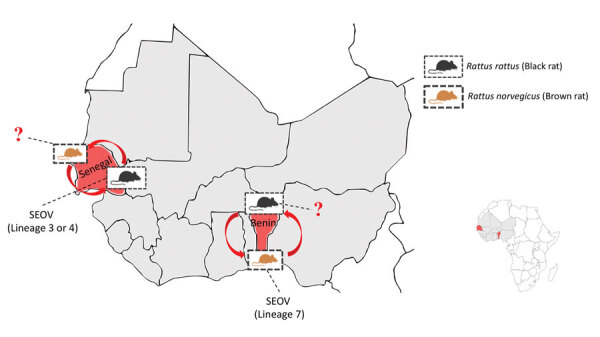
Seaports in which SEOV was detected in rats, West Africa. Detailed map shows localization of the 2 genetically characterized SEOV variants isolated from black rats (*Rattus rattus*) (*18*) and brown rats (*R. norvegicus*) (*19*). Red arrows indicate potential transmission between the rat species. Red question marks indicate current unknown SEOV infection status in the considered rat species. Inset shows the areas of interest on the continent of Africa. SEOV, Seoul orthohantavirus.

## Role of Seaports and Maritime Traffic in Global SEOV Dissemination 

Seaports have already been identified as potential entry points for hantavirus-infected brown rats, suggesting that brown rat–associated SEOV can be readily propagated worldwide through maritime transportation systems (*20*–*22*). In Japan, during the 1960s, brown rats captured in the Tokyo seaport area were shown to have a higher seroprevalence for an HTNV-like agent (*5*). Since then, hantavirus has been detected in rats in other port areas in Asia, including China (*21*,*23*), and in Taiwan, where hantavirus antibody prevalence was much higher (20%) in rodents trapped in international seaports than in rural regions (≈5%), thus suggesting that hantavirus in Taiwan mainly originated from sea transportation (*24*). The role of seaports as the source of hantavirus was further supported by an inverse correlation between the seropositive rate of reservoir host species and the distance of small mammal sampling sites to the seaport (*21*). Of note, SEOV seroprevalence in brown rats from small islands closed to Taiwan was similar to that retrieved in seaports in Taiwan, and the SEOV lineage identified was genetically closely related to SEOV strains from Taiwan. In addition, since 1949, the only channels to trade or travel with those islands has been by boat or airplane to and from Taiwan, pointing again toward the critical role of ship-mediated transportation of rats (rats are more likely transported by boat) in disseminating SEOV in this region (*24*).

From this perspective, Africa is particularly exposed to future introduction events of rodentborne pathogens through maritime traffic due to the exponential increase of trade with the other continents. Increased maritime traffic potentially increases opportunities for ratborne pathogens, particularly SEOV, to expand their geographic range (*18*). Although one third of countries on the continent are landlocked, maritime trade constitutes Africa’s main gateway to international trade with the global marketplace (*25*). Therefore, seaports in Africa can constitute a gateway for allochthonous rodentborne pathogens, notably from Europe and the Americas, the main regions with trading partners, but also from Asia, from which trade has been continuously increasing (*26*). Several rat species are well-known commensals to humans, among which brown rats live in close association with human infrastructure in many countries (*11*). This association could translate into the omnipresence of potential SEOV-carrying brown rats in human-made environments in Africa (*5*). In addition, brown rats can be numerous in seaports located within coastal cities (*27*,*28*), which provides opportunities for local SEOV infection among rats and port workers. Indeed, higher SEOV seroprevalence has been reported in workers in areas where seropositive urban rats were detected (*29*).

## Ratborne Hantavirus Transportation and Spread Via Maritime Traffic

Ratborne hantavirus dissemination through maritime traffic is not a new phenomenon and probably has been occurring since human navigation for migration and trade, involuntarily transporting rodents aboard vessels (*22*). In Madagascar, molecular evidence showed circulation of the variant Anjozorobe virus (ANJZV), belonging to the Thailand hantavirus (THAIV) species, in black rats (*R. rattus*) and in the indigenous Major’s tufted-tailed rat (*Eliurus majori*) (*30*). THAIV is phylogenetically close to but distinct from SEOV, but the 2 viruses share a recent common ancestor (*31*). THAIV is associated with the greater bandicoot rat (*Bandicota indica*) in Thailand (*31*). In addition, THAIV strains Serang and Jurong have been found circulating in Asian house rats (*R. tanezumi*) in Indonesia and Singapore and in Cambodia in *R. rattus* rats (*32*). Detection of the ANJZV variant in Madagascar, far from its most probable areas of origin in South and Southeast Asia, is likely the result of black rat importation into Madagascar through the Arabian Peninsula 2,000–3,000 years ago, when humans colonized the island during a period of vast trading activity in the Indian Ocean (*30*,*33*). Serologic indication of hantavirus circulation in humans also was recently demonstrated in a large national population-based study in Madagascar (*34*), confirming previous observations (*35*). In another study conducted on nearby Mayotte Island, a novel hantavirus, Mayotte virus (MAYOV), which clustered within the THAIV clade, was detected in 18% (29/160) of captured black rats (*36*). That finding also points to ship-transported virus by black rats from Southeast Asia via the Middle East during trade from Arabia thousands of years ago (*30*,*36*).

No available studies describe similar putative human-mediated scenarios for the introduction and spread of hantaviruses within continental Africa. However, SEOV was recently detected in invasive rats in Senegal and Benin (*18*,*19*), suggesting that human-mediated introductions have likely occurred.

## Cross-Transmission from Brown Rats to Other Rodent Species

Although a strong virus–reservoir host specificity is globally accepted for hantaviruses, evidence of interspecies spillover among wild rodents exists, challenging the strict rodent–hantavirus coevolution and giving rise to fears of potential rodent host spectrum expansion (*37*). In Madagascar, the indigenous Major’s tufted-tailed rat was found to be infected by the ANJZV variant, pointing toward a spillover event among rodents from the Muroidea superfamily (*30*). In the same manner, spillover infection is the suspected cause of MAYOV and ANJZV acquisition by *R. rattus* rats from other hantavirus rodent reservoirs in Southeast Asia, such as *B. indica* for THAIV in Thailand and *R. tanezumi* for Jurong and Serang variants in Indonesia (*36*). Another study also showed that, although hantaviruses have preferred host species, spillover events can occur between black rats and domestic mice (*Mus musculus*) (*38*). Natural reassortment has already been documented for SEOV in brown rats and another hantavirus hosted by the striped field mouse (*Apodemus agrarius*) in Asia (*39*). Furthermore, the unambiguous detection of SEOV, both molecularly and serologically, in black rats from Senegal (*18*) shows that SEOV is not restricted to brown rats in Africa and can potentially jump to allied rat species via infected brown rats imported by ship (Figure). This hypothesis has not yet been investigated, but it could have major consequences for SEOV ecology and epidemiology on the continent. Indeed, the brown rat is currently expanding its range across the continent (*40*), which, by itself, might fuel SEOV dissemination in Africa. Even more, SEOV transmission and circulation in black rats could enhance geographic expansion because the *R. rattus* rat species was probably introduced centuries ago (*41*), is already widespread across the continent (*40*), and is still propagating because of its substantial invasive ability (*42*,*43*). When not dominated by other species, black rats are quite numerous in cities and live in close proximity to humans, including within households, especially in socioeconomically and environmentally degraded settlements where rat-to-human zoonotic spillover is possible (G. Dobigny et al., unpub. data, https://doi.org/10.5281/zenodo.6444777). Thus, if *R. rattus* rats are found to be a regular SEOV reservoir, the risk associated with this pathogenic but poorly documented virus in Africa could be even higher than is currently thought.

## Discussion

Because of rapid economic development, environmental and climatic changes, and increased international trade, Africa urgently needs strengthened surveillance and timely rodent elimination in seaport areas, where rats can be numerous, to prevent transmission of rat-associated pathogens and potential disease outbreaks in humans (*22*,*44*). This strategy also represents an efficient way to limit the risk that newly introduced rodentborne viruses might disseminate further across the continent from seaports. To delineate the eco-epidemiology of hantaviruses and their associated risks in Africa, surveillance of viral genetic variability would provide valuable insights into pathogen transmission dynamics among animal reservoirs and the associated disease when human infection occurs. Low intrinsic genetic variability might reflect limited viral evolution and suggest recent colonization events from infected rats arriving via ships from a common source (*20*,*36*). This type of surveillance requires tools available on-site to amplify and characterize viral nucleic acid sequences from hantavirus-infected rodents or patients to unequivocally identify particular variants of SEOV or other hantaviruses, which is not possible with available serologic tests (*45*).

Surveillance in Africa should initially be directed to seaports and seaport workers, which represent the front lines for contamination by newly introduced viruses. However, surveillance is also needed inland because of passive dissemination of the rodent hosts (*22*,*46*), especially if SEOV has already jumped to more widely distributed rodent species. Urban environments might further increase the risk for disease emergence because of close daily contact between humans and rodents, especially rats (*47*; G. Dobigny et al., unpub. data). 

No effective approved hantavirus diseases treatment is available, and whole-virus inactivated vaccines are only licensed for use in South Korea and China but have uncertain protective efficacies (*48*). In addition, only supportive care is available to patients with Seoul virus disease (*9*). Follow-up for rodent biologic invasion, particularly in seaports, is explicitly recommended by the World Health Organization International Health Regulations (2005) (*49*) and is critical for preventing future zoonotic emergence. Thus, seaports could play a role as sentinels of larger surveillance networks.

## Conclusions

Because of associated risk for﻿ animal-to-human spillover of SEOV (*3*), prevention, detection, and healthcare personnel awareness of this often-misdiagnosed infection remain critical on the continent of Africa. Control of rats would require more effective and comprehensive collaboration between local authorities and the academic and research communities. ﻿This type of collaboration fits well with the World Health Organization 13th General Program of Work (*49*). Reducing the reservoir population by using a targeted pest management plan in areas where rodents are highly abundant and in frequent contact with humans could enable mitigation of rodent-related issues and the risk for human disease (K.R. Blasdell et al., unpub. data, https://doi.org/10.1101/2021.03.18.436089). However, ﻿eradication of rat populations in areas of the most concern likely constitutes a more ambitious and unattainable goal and can paradoxically have contrary effects (*50*). Thus, a surveillance rather than riposte-based strategy, combined with medical staff training and implementation of on-site diagnostic methods (*13*), could reduce SEOV outbreak risk among humans in Africa.
